# Integrated analysis of hypoxia-associated lncRNA signature to predict prognosis and immune microenvironment of lung adenocarcinoma patients

**DOI:** 10.1080/21655979.2021.1973874

**Published:** 2021-09-04

**Authors:** Jun Shao, Boqing Zhang, Lin Kuai, Qingguo Li

**Affiliations:** a Department of Cardiothoracic Surgery, The Second Affiliated Hospital of Nanjing Medical University, Nanjing, Jiangsu, China; b Department of Geriatric Medicine, The Second Affiliated Hospital of Nanjing Medical University, Nanjing, Jiangsu, China

**Keywords:** Hypoxia, lncRNA, prognosis, lung adenocarcinoma, TCGA, signature

## Abstract

Lung adenocarcinoma (LUAD) represents the main lung cancer (LC) subtype that possesses a disappointing clinical outcome over the decades. Tumor hypoxia is closely bound up with dismal survival for malignant tumor cases. We identified hypoxia-associated long non-coding RNA (lncRNA) signature to be an explicit indicator for predicting prognosis. The present work acquired RNA-seq and associated clinical data from The Cancer Genome Atlas (TCGA) database. Consensus cluster analysis characterized the hypoxia status of LUAD patients. Cox regression analysis with the least absolute shrinkage and selection operator (LASSO) method determined significantly prognosis-related lncRNAs which were used to create a prognostic model. Diverse statistical approaches like the Kaplan-Meier curve, receiver operating characteristic (ROC) curve, and nomogram were adopted to verify the accuracy of the risk score. The potential immune environment landscape was unearthed by the CIBERSORT algorithm. Three hypoxia-related clusters were determined and 221 differentially expressed hypoxia-related lncRNAs were screened out. We developed a new predictive model based on seven lncRNAs (LINC00941, AC022784.1, AC079949.2, LINC00707, AL161431.1, AC010980.2 and AC090001.1). Kaplan-Meier curves and ROC plots uncovered the reliable predictive power of the risk score model. In addition, the immunosuppressive landscape was presented in the high-risk group by immune cell infiltration analysis. The seven hypoxia lncRNAs survival signature in our article are robust, accurate tools for predicting overall survival in LUAD patients.

## Introduction

Lung cancer (LC) is a frequently seen reason for cancer-associated mortality globally, accounting for the top position in male cancer death and the second position in female cancer death [1]. The most recent data from the National Cancer Registry, Global Cancer Survey 2020, showed that a total of 2,206,771 new cases of lung cancer and 1,796,144 deaths occurred worldwide in 2020 [2]. LC is divided into two types according to histological classification, including non-small cell lung cancer (NSCLC) and small cell lung cancer (SCLC). NSCLC mainly includes lung squamous cell carcinomas (LUSC) and lung adenocarcinoma (LUAD), among them, the incidence of LUAD is the highest [3]. Clinical treatment methods, including surgery, chemoradiotherapy, and targeted therapy have been greatly improved, but the prognosis of LUAD is still unsatisfactory, and the 5-year overall survival (OS) rate is less than 20% [4]. Therefore, it is necessary to exploit newly prognostic biomarkers, which will not only be helpful to improve the prediction of prognosis, but also provide a reference for developing novel therapeutic targets.

Long non-coding RNAs (lncRNAs) are approximately 200 nucleotides in length, which lack protein-coding potential and take up around 70% of non-coding RNAs (ncRNAs) [5]. lncRNAs can interact with mRNAs, microRNAs (miRNAs), DNAs, as well as diverse proteins, which exert vital parts in diverse pathophysiological activities, such as epigenetic regulation, glycolysis, DNA repair, and cell stemness [6–8]. Several reports have indicated that lncRNAs are involved in the regulation of tumor progression through induction of tumorigenesis, invasion, and drug resistance [9]. Currently, numerous lncRNAs have been recognized as lung cancer-associated biomarkers, such as H19, MALAT1, HOTAIR, and JPX [10–13]. Therefore, understanding the effect of lncRNAs in LUAD contributes to determine novel prognostic biomarkers and open up potential therapeutic targets.

Studies have shown that the tumor microenvironment will inevitably appear hypoxic-ischemic state when the diameter of solid tumor exceeds 2 mm. As one of the most obvious characteristics of the tumor microenvironment, hypoxia can activate a variety of intracellular signaling pathways, which subsequently assist in tumor growth, migration, angiogenesis, and apoptosis [14,15]. Accumulating evidence suggests the complex relationship between hypoxia-induced lncRNAs and LUAD. Under hypoxia conditions, AC020978 was proved to be induced in lung cancer cells and facilitate tumor proliferation and glycolytic metabolism through PKM2/HIF axis [16]. Sun et al. reported that CASC15 might be sensitive to hypoxia and enhance cancer cell viability [17]. However, the features of prognostic markers of lung adenocarcinoma based on hypoxia-associated lncRNAs have not been studied. As such, this study was designed to identify hypoxia-related lncRNAs and investigate their clinical potential in LUAD.

The present study intends to classify the different hypoxia statuses of LUAD patients and develop a hypoxia-related lncRNA signature with bioinformatics methods to strengthen the capacity to predict overall survival (OS) of LUAD patients.

## Methods

### Data acquisition

We acquired RNA-seq and associated clinical data from LUAD cases in The Cancer Genome Atlas (TCGA) database. We collected altogether 200 hypoxia-related genes in the hallmark-hypoxia gene set from the Molecular Signatures Database (MSigDB) and are provided in Supplementary Table 1.

### Identification of hypoxia status and differentially expressed hypoxia-related lncRNAs

To characterize the hypoxia status of LUAD patients, we conducted the Consensus Clustering analysis by R package ConsensusClusterPlus according to hypoxia-related genes. The k value (ranging from 2 to 9) was used for determining the best cluster number according to the method of Zhang et. al [18]. Then, we identified the differentially expressed hypoxia-related lncRNAs (HRlncRNAs) with adjusted P-value < 0.05 and |logFC| ≥ 1 between two candidate clusters by *limma* package in R [19].

### Construction and verification of the prognosis signature associated with hypoxia status

For improving the risk score creditability, we classified all LUAD cases as training and test sets at the ratio of 1:1. Of them, the training set was used to construct a prognosis prediction model, whereas the entire set and test set were adopted to validate the prediction performance. First of all, significant prognostic lncRNAs were identified by univariate Cox hazard regression based on DEHRlncRNAs in the training set. Later, the R package *glmnet* function was employed to conduct Cox regression and LASSO regression. Afterward, multivariate Cox regression analysis was employed to construct the risk signature for predicting the prognosis in LUAD patients. The risk score was calculated as following in a formula: (HRlncRNA 1 expression × coefficient) + (HRlncRNA 2 expression × coefficient) + … + (HRlncRNA n expression × coefficient). At the same time, the cases were classified into low- or high-risk groups based on the median value of risk score. In addition, the entire set and test set were used to validate our signature.

### Construction of signature-based nomogram

After the collinearity test, risk score and related clinical parameters were included to construct a predictive nomogram [20]. The 1 -, 3 -, and 5-year OS of LUAD patients in the whole TCGA set were predicted by nomogram. Subsequently, the accuracy of the prognostic nomogram will be verified according to the correction chart of predicted survival rate and observed survival rate.

### Gene set enrichment analysis (GSEA)

GSEA was adopted to check the high-risk associated pathways or biological functions. This study employed GSEA software to analyze those expressed genes between high- and low-risk groups and the Hallmark gene set collected from the Molecular Signatures Database v7.1. In line with the GSEA user guide, NOM P < 0.05 and | NES | > 1 were considered significant [21].

### Infiltrating immune cells analysis of the prognostic signature

The landscape of immunocyte infiltration in LUAD samples was achieved from the leukocyte gene matrix of CIBERSORT. By performing the CIBERSORT algorithm, we examined the proportion of 22 immunocyte subtypes between low- and high-risk cohorts.

### Clinical specimen collection

A total of twenty pairs of LUAD tissues and corresponding adjacent normal tissues were collected from the Second Affiliated Hospital of Nanjing Medical University (NJMU), all of which were pathologically diagnosed as LUAD. All excised tissue samples were immediately stored in liquid nitrogen until required. This study was approved by the Institutional Review Board and the Ethics Committee of the Second Affiliated Hospital of NJMU, and informed consent was obtained from all patients with LUAD.

### Cell culture

Two human LUAD cell lines (A549 and NCI-H460) and one human lung epithelial cell line (BEAS-2B) were purchased from the Shanghai Institute of Biochemistry and Cell Biology, the Chinese Academy of Sciences. All cell lines were cultured in RPMI 1640 medium containing 10% fetal bovine serum (FBS, Gibco Company) and 1% antibiotics (100 U/ml penicillin G and 100 mg/ml streptomycin) at 37°C with 5% CO2.

### RNA extraction and quantitative Real-Time Polymerase Chain Reaction (qRT-PCR)

Total cellular RNA was extracted from cells by Trizol (Vazyme biotech, Nanjing, China). Then, we used a BioSpec-nano spectrophotometer (Shimadzu, Japan) to measure the concentration and purity of extracted cellular RNA. Prime Script RT Master Mix reagent (Takara Bio, Dalian, China) was used to synthesize complementary DNA (cDNA). Next, we used qRT-PCR using the StepOnePlus real-time PCR system (Thermo Fisher Science) with polymerase chain reaction system TB Green®PreMix Ex Taq™ (Takara Bio, Dalian, China) and 2^−ΔΔCT^ method to calculate the relevant gene expression. The particular primers are listed in **Supplementary Table 2**. GAPDH was used as the internal control for lncRNA expression and U6 for miRNAs expression.

### Cell transfection

In this study, siRNA negative control (si‐NC) and si‐AL161431.1 were chemically synthesized by Ribobio (Guangzhou, China). The sense sequence of si- AL161431.1 was 5ʹ-GUUUCCUGAACUUUAAUGATT-3ʹ. Then, Lipofectamine 3000 (Invitrogen, CA, USA) was utilized to transfect LUAD cells with siRNAs in line with the manufacturer’s protocol. At 48 h post-transfection, we harvested cells for *in vitro* analysis.

### Cell counting Kit-8 (CCK-8) assay

LUAD cells (2000/well) were seeded into the 96-well plates and cultured within RPMI-1640 that contained 10% FBS. At a fixed time of day, we added CCK8 solution into each well to incubate cells under 37°C for an additional 2 h. The absorbance value was measured at 450 nm by a microplate spectrophotometer (Thermo, USA) and was utilized to detect the capability of tumor cell proliferation.

### Colony formation assay

SiRNA-transfected cells (300/well) were plated into the 6-well plates and cultured for 10 days within the RPMI-1640 medium that contained 10% FBS. Later, 1% formaldehyde was adopted to fix proliferating cell colonies, whereas 1% crystal violet was applied in staining.

### Transwell assay

The Transwell chamber (pore size, 8 μm; Corning Costar Corp, USA) was used to detect cell migration. After suspending the stably transfected LUAD cells into the serum-free RMPI-1640 medium, the upper chamber was added with cell suspension. Afterward, the RMPI-1640 medium (500 μl) that contained 10% FBS was placed into the lower chamber, followed by 24 h co-culture under 37°C. Later, we removed cells on the upper membrane surface with cotton swabs and further stained cells on the lower surface of the membrane with 1% crystal violet.

### Apoptosis analysis

Cell apoptosis was analyzed with a cell apoptosis detection kit (Vazyme, Nanjing, China). The transfected cells were washed with phosphate-buffered saline (PBS), resuspended in 500 μl of binding buffer, and stained with 5 μl of propidium iodide (PI) and 5 μl of annexin V-FITC solution. Then, CytoFLEX Flow Cytometer (Beckman, USA) was used to detect the cells.

### Luciferase Reporter Assay

The target sequences of lncRNA AL161431.1 containing wild-type or mutant-binding site of miR-1252-5p were subcloned into pmirGLO vector (Promega, Madison, USA) to form wt-AL161431.1 or mut-AL161431.1, respectively. Next, AL161431.1-wt/ AL161431.1-mut and miR-1252-5p/miR-NC were co-transfected into A549 cells. After 48 h, luciferase activity was examined by using a Dual-Luciferase Reporter Kit (Solarbio, China).

### Statistical analysis

R software v3.6.3 and GraphPad Prism v8.01 were applied in statistical analyses. mRNA expression, immune cell infiltration score, and pain risk were compared between the two groups through the Wilcox test. One-way ANOVA was utilized to analyze continuous variables among different groups. Independent prognostic analysis was conducted by univariate as well as multivariate Cox regression. The difference in survival of high- and low-risk groups was analyzed by Kaplan-Meier (K-M) survival curve. In addition, we plotted receiver operating characteristic (ROC) curves for assessing the sensitivity and specificity of our constructed prognosis model. P < 0.05 stood for statistical significance.

## Results

In the current study, we intended to characterize the different hypoxia states and screen HRlncRNAs in LUAD. based on the integrated analysis of TCGA-LUAD dataset, we created a novel HRlncRNAs signature and nomogram to accurately predict prognosis of LUAD cases. Immunity relative analysis and functional enrichment annotation were used to explore the clinical potency and underlying mechanisms of our hypoxia-based risk signature. Moreover, the lncRNA AL161431.1 was selected to verify our signature by in vitro analyses.

### Consensus clustering determined hypoxia-related clusters of LUAD

**Figure 1.**
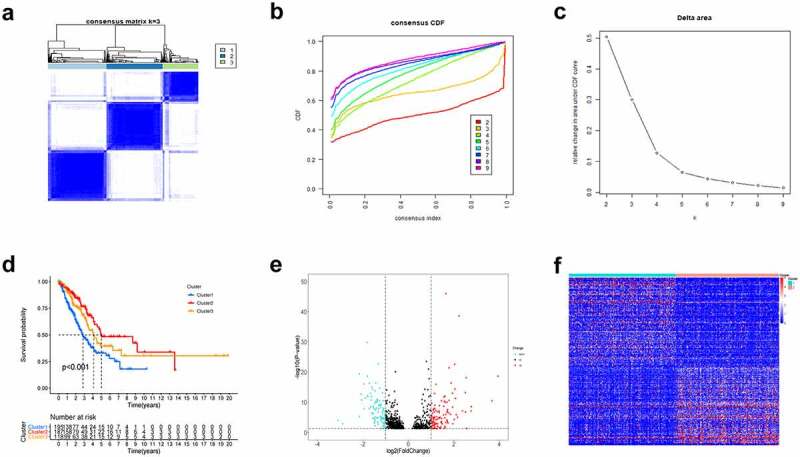
Consensus Clustering identified hypoxia-related clusters of LUAD samples. (a) Consensus matrix for k = 3. (b) The CDF value of consensus index. (c) Relative change in area under CDF curve. (d) Kaplan–Meier OS survival curves for three clusters. Volcano plot (e) and heatmap (f) identifying differentially expressed hypoxia-related lncRNAs

Based on the expression profile matrix of 200 hypoxia-related genes, we utilized the Consensus Clustering Method to explore the hypoxia status by clustering LUAD cases. When k value = 3, the unsupervised clustering was most stable and three clusters named C1 (n = 195), C2 (n = 187) and C3 (n = 118) were generated (Figure 1(a-c)). Furthermore, we used survival analysis to investigate the relationship between hypoxia status and prognosis of LUAD patients. The results showed that cases of C2 exhibited the best prognosis, whereas patients of C1 had the worst OS (Figure 1(d)).

### Identification of the differentially expressed HRlncRNAs

Based on the lncRNA expression levels of the C1 cluster and C2 cluster, a total of 221 differentially expressed HRlncRNAs were identified by *limma* package with |logFC| ≥ 1 and adjusted P-value < 0.05 (Figure 1(e)). Heatmap characterized the HRlncRNA pattern in LUAD samples (Figure 1(f)).

### Establishment of HRlncRNAs prognosis signature and survival analysis

**Figure 2.**
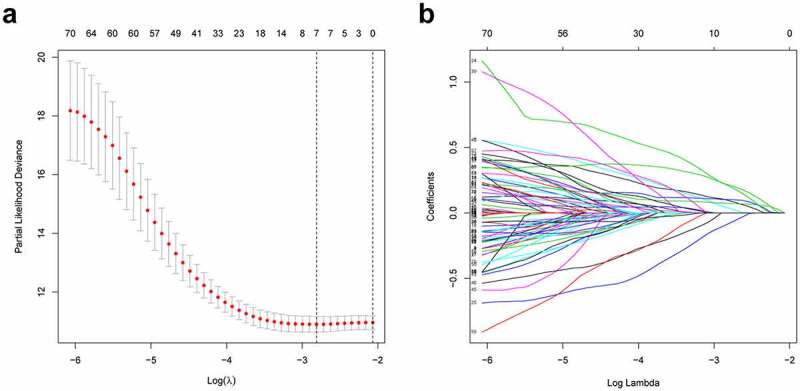
Construction of prognostic risk signature based on hypoxia-related lncRNAs. (a) LASSO coefficient profiles of the 79 lncRNAs in the training cohort. (b) Cross-validation for tuning the parameter selection in the LASSO analysis

**Figure 3.**
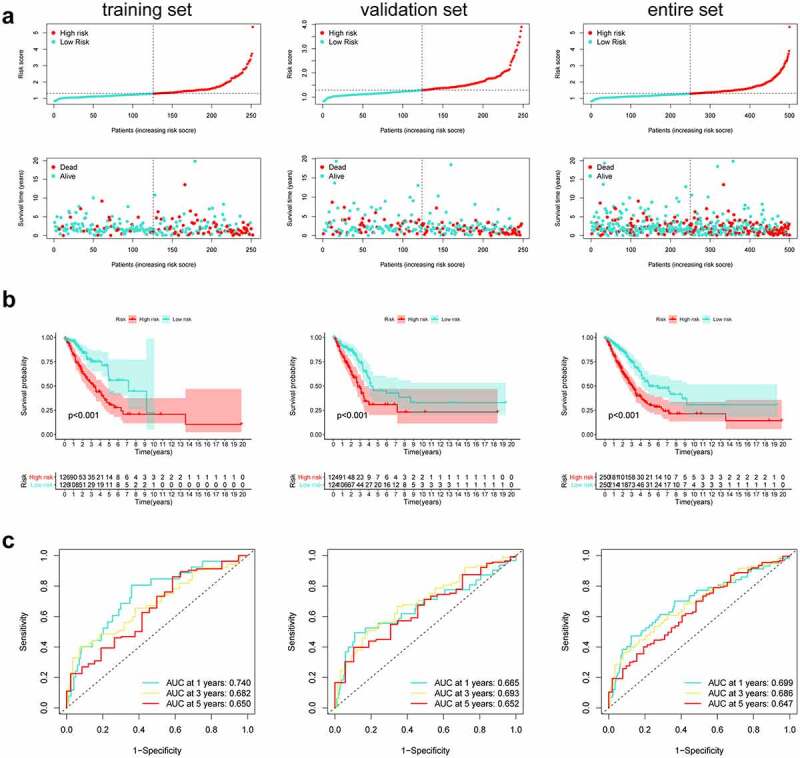
Predictive characteristics of the seven hypoxia-related lncRNAs signature. (a) Distribution of risk scores and survival status of high- and low-risk patients. (b) K-M analyses for both risk groups. (c) ROC curve analysis for verifying model performance in the prediction of LUAD survival rates at 1, 3, and 5 years

Firstly, according to the ratio of 1:1, the TCGA entire set (n = 504) were split into training group (n = 252) and validation group (n = 252). Univariate Cox regression was conducted to analyze the correlation between 79 PRlncRNAs and OS in the training set. For reducing the overfitting risk, ‘glmnet’ package was used for LASSO-Cox regression (Figure 2). Finally, by performing multivariate Cox regression method, a prognostic hypoxia-related risk model composed of seven lncRNAs (LINC00941, AC022784.1, AC079949.2, AC090001.1, LINC00707, AL161431.1, and AC010980.2) was set up (Table 1). This formula was adopted to generate the risk score: [LINC00941 expression × (0.0954)] + [AC022784.1 expression × (0.0378)] + [AC079949.2 expression × (0.2147)] + [AC090001.1 expression × (−0.0828)] + [LINC00707 expression × (0.1019)] + [AL161431.1 expression × (0.0740)] + [AC010980.2 expression × (0.2083)]. All cases were classified as high- or low-risk groups according to the median risk score. Figure 3 illustrated the predictive power of the prognostic model. Significantly, as revealed by KM curves, the high-risk group had markedly reduced OS compared with the low-risk group (Figure 3(b)). This study also utilized ROC analysis for assessing the prediction performance of the selected prognostic markers. It was seen from Figure 3 that, the area under the ROC curves (AUC) values for 1-, 3-, and 5-year OS were 0.740, 0.682, and 0.650, respectively, for the training set. Meanwhile, this study verified the above findings in both the test and entire sets (Figure 3(c)).

**Table 1.** Seven hypoxia-related prognostic lncRNAs significantly associated with OS

lncRNA	Coefficient	Hazard ratio (95% CI)	P-value
LINC00941	0.0954	1.65 (1.39–1.95)	0.113
AC022784.1	0.0378	1.34 (1.19–1.52)	0.003
AC079949.2	0.2147	1.56 (1.27–1.91)	0.277
AC090001.1	−0.0828	0.61 (0.42–0.88)	0.023
LINC00707	0.1019	1.57 (1.34–1.85)	0.046
AL161431.1	0.0740	1.30 (1.16–1.46)	<0.001
AC010980.2	0.2083	1.53 (1.20–1.95)	<0.001

### Construction and validation of a prognostic nomogram

**Figure 4.**
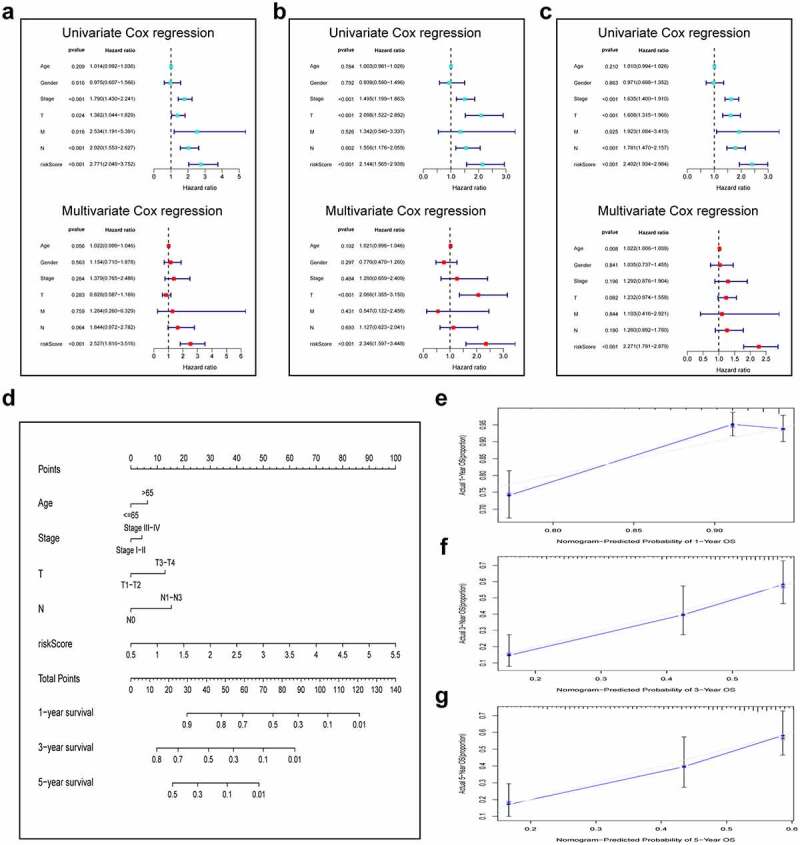
Integration of HRlncRNAs and clinical characteristics in predicting LUAD prognosis. (a-c) univariate analysis and multivariate analysis containing risk score and clinical factors. (d) Nomogram constructed to predict OS rates at 1, 3, and 5 years. The nomogram calibration curves on consistency between predicted and observed (e) 1-, (f) 3-, and (g) 5-year survival

For validating the performance of our constructed prognosis prediction model in predicting OS of LUAD cases, univariate as well as multivariate Cox regression was conducted. Risk score (P < 0.001), T stage (P = 0.024), N stage (P < 0.001), M stage (P = 0.016), and clinical stage (P < 0.001) were identified as the independent factors to predict the unfavorable OS for LUAD cases in the training set, as shown by univariate Cox regression. Then, the significance of the risk score (P < 0.001) was further proved in the multivariate Cox analysis (Figure 4(a)). The same results were confirmed in the validation set and the entire cohort. (Figure 4(b,c)). In addition, to optimize the prediction performance of our constructed prediction model, the risk score was used in combination with other clinicopathological parameters to develop a new nomogram for predicting clinical outcome at 1, 3, and 5 years in LUAD patients (Figure 4(d)). The calibration curves exhibited no deviations between the ideal line predicted by the nomogram and the actual survival rate line (Figure 4(e-g)).

### Functional analysis of the seven-lncRNAs prognosis model

**Figure 5.**
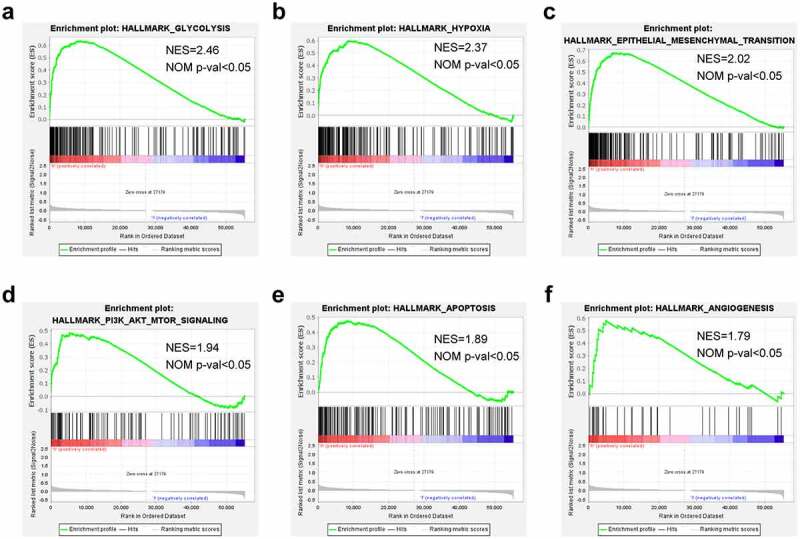
Gene set enrichment analysis demonstrating hallmarks for the risk signature. (a) GSEA on glycolysis, (b) GSEA on hypoxia, (c) GSEA on epidermal-mesenchymal transition, (d) GSEA on PI3K/AKT/MTOR signaling, (e) GSEA on apoptosis, (f) GSEA on angiogenesis

To delineate the cancer hallmarks and their corresponding functions of the seven-lncRNAs signature involved in LUAD progression, this study conducted GSEA on high- and low-risk groups. The funding revealed that ‘glycolysis’, ‘hypoxia’, ‘epidermal-mesenchymal transition’, ‘PI3K-AKT-MTOR signaling’, ‘apoptosis’, and ‘angiogenesis’ were all in a significant activation state in high-risk patients. In summary, high risk was closely related to the process of stimulating tumor proliferation and anti-apoptosis (Figure 5).

### Immune environment landscape between high and low-risk patients

**Figure 6.**
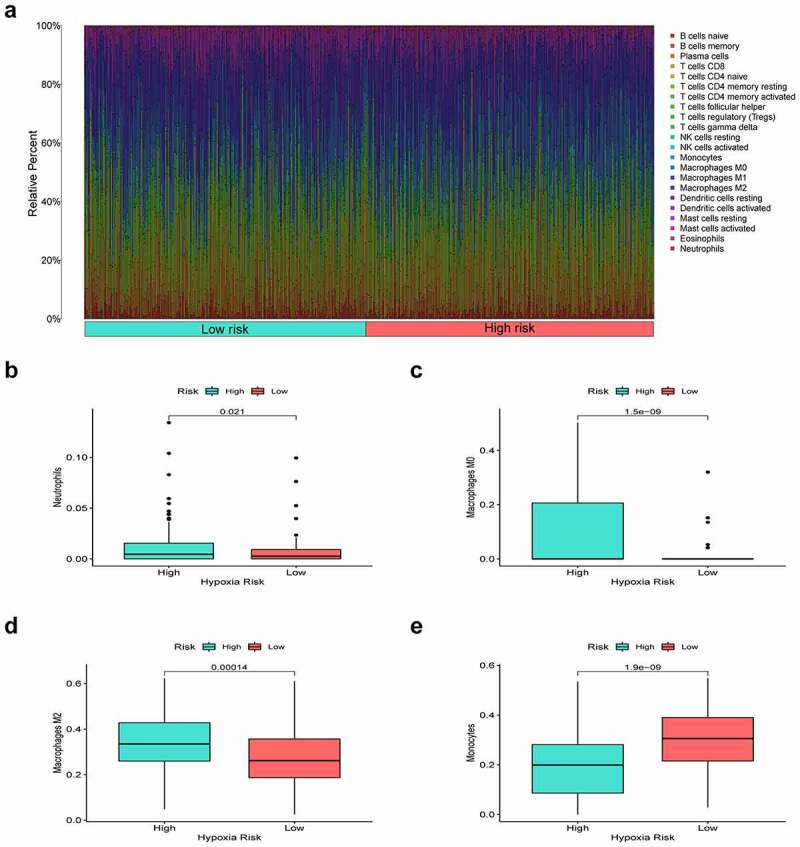
Immune environment landscape between high and low-risk patients. (a) Barplot illustrating the proportion of immune cell infiltration in high and low-risk groups. (b-e) Box plots showing significantly different immune cells between high-risk and low-risk groups

**Figure 7.**
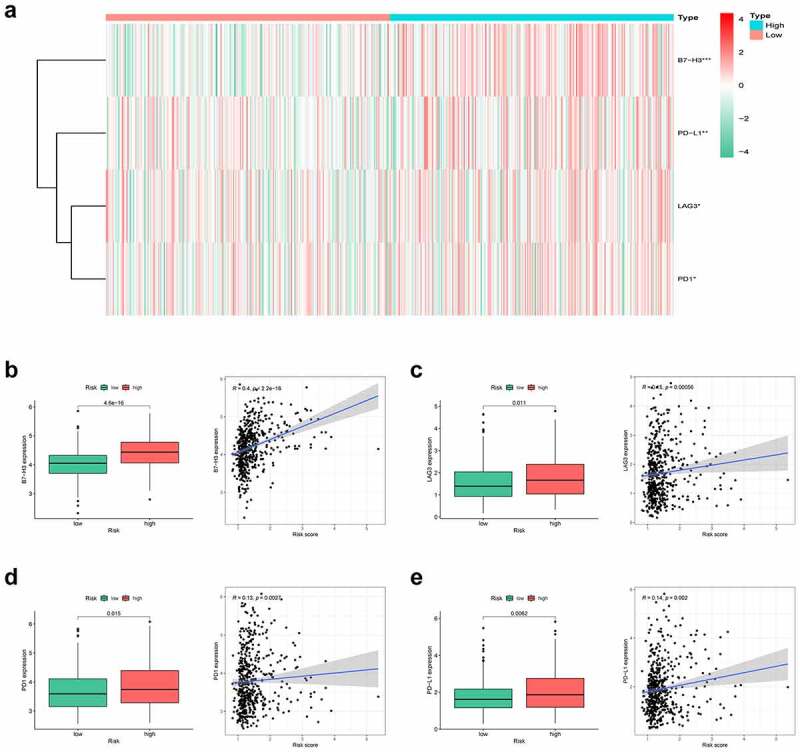
Correlation analysis of risk group and immune checkpoints. (a) Heatmap of the immune checkpoint expression profiles in high-risk and low-risk groups. (b) B7H3, (c) LAG3, (d) PD1, (e) PD-L1

Hypoxia is a noteworthy factor of immune escape, so we further used CIBERSORT analysis to mirror the status of immune cells. Here, we evaluate the differences in the immune cells between two risk subgroups. Figure 6(a) reflected the immune cell landscape between two risk subgroups. As a result, neutrophils, M0 macrophages, and M2 macrophages showed higher levels in the high-risk group, while Monocytes remarkably showed higher levels in the low-risk group (Figure 6(b-e)). Interestingly, we also found that the proposed signature was closely related to immune checkpoints (Figure 7). Both two-sample T-test and Pearson correlation analysis proved that the higher the risk score, the higher the expressions of the key immune checkpoints (B7-H3, LAG3, PD-1, and PD-L1).

### Restriction of AL161431.1 weakened LUAD cell proliferation, migration, and induced apoptosis

**Figure 8.**
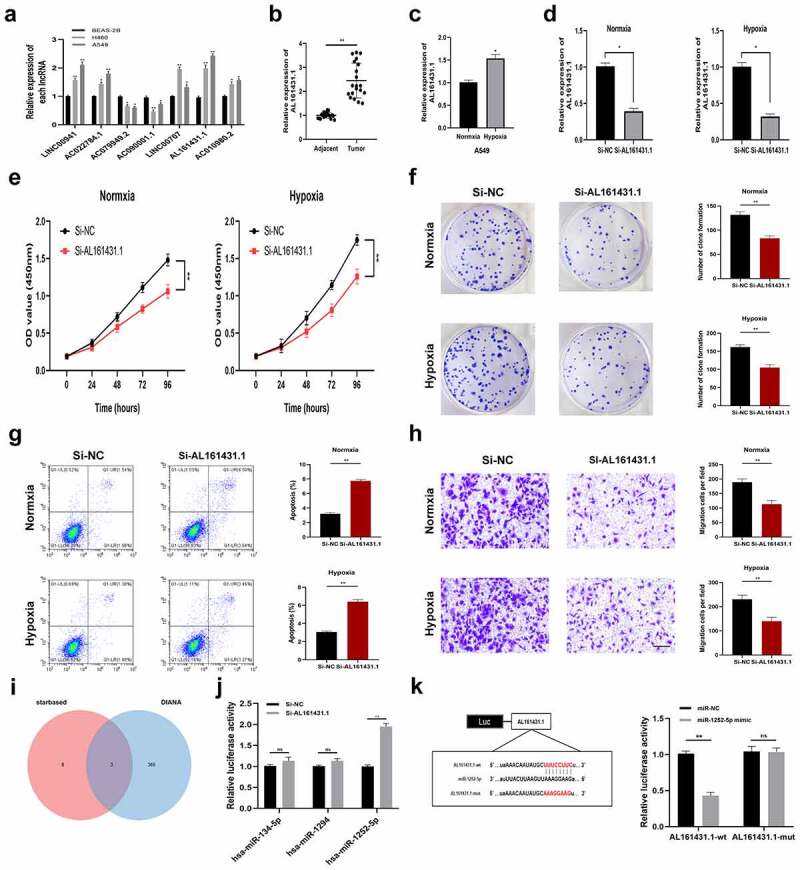
Inhibiting the expression of AL161431.1 blocks LUAD cell proliferation. (a) The mRNA expression level of seven signature lncRNAs in BEAS-2B and two LUAD cell lines (b) Relative expression of AL161431.1 in LUAD tissues and adjacent normal tissues. (c) AL161431.1was upregulated in A549 cells by hypoxia treatment. (d) AL161431.1 was inhibited in A549 using siRNAs. The effect of AL161431.1 on proliferation in A549 was detected using CCK-8 (e) and colony formation assays (f). (g) Transwell assays for cell migration (Scale bars: 100 μm). (h) Flow cytometric analysis of A549 cells transfected with siRNAs or si-NC about apoptotic rates. (i) Venn diagram showed the downstream target miRNAs of AL161431.1 by starbase and DIANA database. (j) The expression of three predicated miRNAs by qRT-PCR. (k) Luciferase assay in A549 cells after co-transfected either AL161431.1-wt or AL161431.1-mut vectors with miR-1252-5p mimic (*p < 0.05; **p < 0.01; ***p < 0.001)

First, we analyzed the expression levels of seven lncRNAs in BEAS-2B, H460, and A549 by a qRT-PCR assay (Figure 8(a)). Since AL161431.1 exhibited the most marked differentiation between BEAS-2B and LUAD cells (H460, A549), so we selected AL161431.1 to verify our signature in the next study. As shown in Figure 8(b), the qRT-PCR analysis demonstrated that AL161431.1 showed higher expression within LUAD tissues relative to adjacent normal tissues. Then, we used A549 cells to carry out the *in vitro* experiments. Intriguingly, the expression of AL161431.1 was increased by hypoxia treatment (Figure 8(c)). Figure 8(d) indicated the good knockdown efficiency of si-AL161431.1 transfection. According to CCK8 assays, AL161431.1 silencing markedly suppressed LUAD cell proliferation under normxia and hypoxia conditions (Figure 8(e). Conforming to CCK8 assay results, colony formation experiments revealed that knockdown of AL161431.1 suppressed the proliferation of A549 cells (Figure 8(f)). Apoptosis assay was used to further clarify the mechanism by which AL161431.1 inhibited cell proliferation. The result showed that the apoptotic rate was increased in the si-AL161431.1 group (Figure 8(g)). We also found that downregulation of AL161431.1 significantly blocked the migration of A549 cells (Figure 8(h)). To explore the downstream mechanism of AL161431.1, we obtained three potential miRNAs (hsa-miR-134-5p, hsa-miR-1294, and hsa-miR-1252-5p) with a high binding score by online tool starbase and DIANA (Figure 8(i)). Next, the qRT–PCR assay showed that only miR-1252-5p was negatively regulated by AL161431.1 (Figure 8(j)). Additionally, dual-luciferase reporter assays proved that AL161431.1-related luciferase activity was significantly inhibited by overexpressing miR-1252-5p (Figure 8(k)).

## Discussion

Lung cancer ranks the frequently occurring reason for cancer-associated mortality globally, and the mortality and incidence rate is the highest among malignancies, of which 85% are NSCLC [22]. With the continuous development of the anti-smoking campaign, the cases of LUAD gradually dominates in NSCLC. LUAD has generally progressed to intermediate and advanced stages at the time of diagnosis, losing the best time for radical surgery, and the median survival of patients with intermediate and advanced stages is only 10–11 months after radiotherapy and chemotherapy [23]. The hypoxic niche in the tumor is the source of metastasis and resistance to treatment, and it accounts for a major reason for treatment failure. Although IncRNAs are increasingly regarded as biomarkers for predicting OS of various cancers, prognostic indicators based on the hypoxic lncRNAs are still largely unexplored in LUAD. Therefore, it is essential to find hypoxia-related molecules to determine the effective prognostic biomarkers of LUAD. Here, we established a reliable HRlncRNAs based prognostic model and confirmed its clinical application in LUAD patients. Furthermore, we also preliminarily explore the carcinogenic effect of AL161431.1 in LUAD cells and found that inhibiting AL161431.1 can block the proliferation of tumor cells.

In this article, we first classified the LUAD samples into three hypoxia states (C1, C2, and C3) according to the hypoxia-associated genes expression profiles. Patients in the C1 cluster had markedly reduced OS compared with C2. Afterward, we analyzed the 221 DEHRlncRNAs between the two above-mentioned groups for constructing the lncRNAs-based prognosis prediction model. A novel hypoxia-related lncRNAs model was generated through Cox regression analysis as well as the Lasso regression method in the TCGA training set. Next, using the constructed risk model, we calculated the risk scores for all LUAD patients and classified them as a high- or low-risk group. According to Kaplan-Meier analysis, cases having low-risk scores had superior OS to those having high-risk scores. In addition, the AUC value of ROC plots illustrated the reliability and accuracy of the prognostic model. We further verified that the model could be the factor to independently predict the prognosis upon univariate as well as multivariate Cox regression. Meanwhile, these results were also confirmed in the TCGA validation set and the entire set. Moreover, we predigested the risk model and integrated other clinical-related factors to set up a nomogram that generates a score representing the prognosis of LUAD. The calibration curves demonstrated the satisfactory predictive ability of the constructed nomogram.

Seven key lncRNAs derived from our model were distinctly correlated with OS in patients with multiple tumors. Among these seven lncRNAs, only AC090001.1 is a potential protective factor, while LINC00941, AC022784.1, AC079949.2, LINC00707, AL161431.1, and AC010980.2 are all potential risky indicators. Before this, all the risky lncRNAs already have been confirmed to be linked to LUAD. Ren et al. analyzed LINC00941 expression within the LUAD and non-carcinoma tissue samples and clarified that high LINC00941 expressions could accelerate tumor progression and angiogenesis, which offers new insights into comprehension and targets for LUAD diagnosis and treatment [24]. The metastatic and proliferative performance of LINC00941 also proved in pancreatic adenocarcinoma, colon cancer, papillary thyroid cancer, and gastric cancer [25–28]. Our results are in strong agreement with these researches, indicated that LINC00941 is positively related to the worse prognosis of patients. LINC00707, the miR-876 molecular sponge, modulates MTDH expression within breast cancer [29]. The abnormal LINC00707 expression is reported to regulate cisplatin sensitivity via sponging miR-145 in NSCLC cells [30]. In endometrial carcinoma, AL161431.1 acts as an oncogene to promote cell proliferation and migration through the MAPK signaling pathway [31]. By constructing a survival-related ceRNA network, the investigators found that AL161431.1 had significant predictive value for overall survival in LUSC patients [32]. In addition, AC079949.2, AC022784.1, and AC010980.2 were identified to build risk signature for improving the prediction of LUAD prognosis [33–35]. However, AC090001.1 has not been previously reported in any cancers.

An increasing body of evidence indicates that hypoxia mediates immunosuppression by boosting suppressive immune cells (TAMs, Tregs, and neutrophils) and inducing immune checkpoints in the tumor environment [36]. In terms of mechanism, cancer cells can release various chemokines and subsequently recruit monocytes in the tumor environment. Within the tumor tissue, the recruited monocytes swiftly TAMs which exhibit the low ability of antigen presentation and suppresses T cell growth and activation [37]. It has been reviewed that TAMs were enriched in hypoxic regions and secrete cytokines resulting in tumor proliferation and angiogenesis [38,39]. Furthermore, crucial funding proved that TAM can be a robust predictor of poor prognosis for lung cancer patients [40].

According to our findings, M2 macrophage infiltration showed a positive correlation with the hypoxia risk, which was in line with the dismal prognosis of high-risk cases. Besides, neutrophils, another pivotal immunosuppressive cell, increased in the high-risk group, which suggested that the high-risk group was associated with the immunosuppressive microenvironment. The tumor hypoxia can also protect tumors from immune surveillance by regulating the expression of immune checkpoint molecules. More specifically, hypoxia‐inducible factor‐1 (HIF‐1) could selectively stimulate the expression of PD-L1 and PD-L2 on myeloid‐derived suppressor cells (MDSCs) or macrophages [41]. Our results showed that B7-H3, LAG3, PD-1, and PD-L1 were significantly increased in high-risk patients indicating that the target of these immune checkpoints might enhance the efficacy of immunotherapy for LUAD patients.

Finally, we explored the functional significance of the AL161431.1 in LUAD cells by experimental studies. The expression level of AL161431.1 was upregulated under the hypoxia condition. *In vitro* analysis showed that inhibition of AL161431.1 limited cell proliferation and migration and induced apoptosis in A549 cells. Furthermore, we found that AL161431.1 might exert a ‘sponge-like’ function by binding with miR-1252-5p.

Certain limitations must be noted in the present work. First, the prognosis prediction model was just constructed based on TCGA datasets, while validation by external dataset was lacking. Secondly, the relationship between lncRNA expression and immune cell infiltration and underlying molecular mechanism of lncRNAs in LUAD needs further *in vitro* and *in vivo* experimental studies.

## Conclusion

In summary, we discriminated against LUAD patients with different hypoxia statuses and built a seven hypoxia-related lncRNAs signature which largely improved the prognosis of LUAD patients and reflected the immune environment. Our risk signature might benefit precision treatment for LUAD.

## Supplementary Material

Supplemental Material

## Data Availability

Publicly available datasets were analyzed in this study. These data can be found here: TCGA (https://portal.gdc.cancer.gov/).
