# Supplementary material for: Integrated analysis of hypoxia-associated lncRNA signature to predict prognosis and immune microenvironment of lung adenocarcinoma patients
Source: Bioengineered. 2021 Sep 4;12(1):6186–200. doi: 10.1080/21655979.2021.1973874 (PMC8806605; doi:10.1080/21655979.2021.1973874)
Supplement: Supplemental Material [file KBIE_A_1973874_SM6320.zip › supplementary/Supplementary Table 1.docx]

**Table 1** Seven hypoxia-related prognostic lncRNAs significantly associated with OS.

| **lncRNA** | **Coefficient** | **Hazard ratio (95% CI)** | **P-value** |
| --- | --- | --- | --- |
| LINC00941 | 0.0954 | 1.65 (1.39-1.95) | 0.113 |
| AC022784.1 | 0.0378 | 1.34 (1.19-1.52) | 0.003 |
| AC079949.2 | 0.2147 | 1.56 (1.27-1.91) | 0.277 |
| AC090001.1 | -0.0828 | 0.61 (0.42-0.88) | 0.023 |
| LINC00707 | 0.1019 | 1.57 (1.34-1.85) | 0.046 |
| AL161431.1 | 0.0740 | 1.30 (1.16-1.46) | <0.001 |
| AC010980.2 | 0.2083 | 1.53 (1.20-1.95) | <0.001 |
