# Supplementary material for: Integrated analysis of hypoxia-associated lncRNA signature to predict prognosis and immune microenvironment of lung adenocarcinoma patients
Source: Bioengineered. 2021 Sep 4;12(1):6186–200. doi: 10.1080/21655979.2021.1973874 (PMC8806605; doi:10.1080/21655979.2021.1973874)
Supplement: Supplemental Material [file KBIE_A_1973874_SM6320.zip › supplementary/Supplementary Table 2.docx]

| **Gene** | **Sequence** | |
| --- | --- | --- |
| LINC00941 | Forward | 5′- GACCTTTTCAGGCCAGCATT -3′ |
|  | Reverse | 5′- ACAATCTGGATAGAGGGCTCA -3′ |
| LINC00707 | Forward | 5′- TCACATCTGTGAAAAGAGTGCT-3′ |
|  | Reverse | 5′- CTGGACTGTGAGTACCAGGC -3′ |
| AC010980.2 | Forward | 5′- CCACAGTGCTCGGCTAAAGA -3′ |
|  | Reverse | 5′- CTTTGTTTTCTCGCTGCGCT -3′ |
| AC022784.1 | Forward | 5′- GUUUCCUGAACUUUAAUGATT-3′ |
|  | Reverse | 5′-UCAUUAAAGUUCAGGAAACTT-3′ |
| AL161431.1 | Forward | 5′- CTTTGAGCAAGGTCCGCAAG -3′ |
|  | Reverse | 5′- AGGTACCACAGGAGGCACAA -3′ |
| AC090001.1 | Forward | 5′- GTCTTGTTCTGCTACCCTCCA -3′ |
|  | Reverse | 5′- GCTCCACATTCACTTTCCATA -3′ |
| AC079949.2 | Forward | 5′- AATCGATACTCGGTTGCGGT -3′ |
|  | Reverse | 5′- TGGTCAGGAGCTAATGGGGA -3′ |
| miR-134-5p | Forward | 5′- CCGCTCGAGCCGGCCTTCCAACCTTTGTC -3′ |
|  | Reverse | 5′- GAATGCGGCCGCTCCCATCATCAATATTTATTG -3′ |
| miR-1294 | Forward | 5′- ACACTCCAGCTGGGTGTGAGGTTGGCATTG-3′ |
|  | Reverse | 5′-CTCAACTGGTGTCGTGGAGTCGGCAATTCAGTTG-3′ |
| miR-1252-5p | Forward | 5′- TGTGAGGTGTAGAAAGAAGGAA -3′ |
|  | Reverse | 5′- ATTAAGCTCATTTGGAATTCTCT -3′ |
| U6 | Forward | 5′-TGCGGGTGCTCGCTTCGGC-3′ |
|  | Reverse | 5′-CCAGTGCAGGGTCCGAGGT-3′ |
| GAPDH | Forward | 5′- GTCTCCTCTGACTTCAACAGCG -3′ |
|  | Reverse | 5′- ACCACCCTGTTGCTGTAGTAGCCAA -3′ |

**Supplemental Table 2. The primer sequences of each gene in this study.**
